# Epidemiological characteristics of COVID-19 cases and estimates of the reproductive numbers 1 month into the epidemic, Italy, 28 January to 31 March 2020

**DOI:** 10.2807/1560-7917.ES.2020.25.49.2000790

**Published:** 2020-12-10

**Authors:** Flavia Riccardo, Marco Ajelli, Xanthi D Andrianou, Antonino Bella, Martina Del Manso, Massimo Fabiani, Stefania Bellino, Stefano Boros, Alberto Mateo Urdiales, Valentina Marziano, Maria Cristina Rota, Antonietta Filia, Fortunato D'Ancona, Andrea Siddu, Ornella Punzo, Filippo Trentini, Giorgio Guzzetta, Piero Poletti, Paola Stefanelli, Maria Rita Castrucci, Alessandra Ciervo, Corrado Di Benedetto, Marco Tallon, Andrea Piccioli, Silvio Brusaferro, Giovanni Rezza, Stefano Merler, Patrizio Pezzotti, the COVID-19 working group, COVID-19 working group, COVID-19 working group, Angela di Martino, Marzia Facchini, Stefania Giannitelli, Fabiola Mancini, Simona Puzelli, Roberta Urciuoli, Antonia Petrucci, Michele Labianca, Anna Domenica Mignuoli, Angelo D'Argenzio, Erika Massimiliani, Tolinda Gallo, Paola Scognamiglio, Camilla Sticchi, Danilo Cereda, Daniel Fiacchini, Francesco Sforza, Maria Grazia Zuccaro, Pier Paolo Benetollo, Donatella Tiberti, Maria Chironna, Maria Antonietta Palmas, Salvatore Scondotto, Emanuela Balocchini, Anna Tosti, Mauro Ruffier, Filippo Da Re

**Affiliations:** 1 Istituto Superiore di Sanità, Rome, Italy; 2 These authors contributed equally; 3 Bruno Kessler Foundation, Trento, Italy; 4 Department of Epidemiology and Biostatistics, Indiana University School of Public Health, Bloomington, United States; 5 Laboratory for the Modeling of Biological and Socio-technical Systems, Northeastern University, Boston, United States; 6 Cyprus University of Technology, Limassol, Cyprus; 7 European Programme for Intervention Epidemiology Training (EPIET), European Centre for Disease Prevention and Control (ECDC), Stockholm, Sweden; 8 The members of the COVID-19 working group are listed under Investigators

**Keywords:** COVID-19, SARS-CoV-2, descriptive epidemiology, infectious disease modelling

## Abstract

**Background:**

On 20 February 2020, a locally acquired coronavirus disease (COVID-19) case was detected in Lombardy, Italy. This was the first signal of ongoing transmission of severe acute respiratory syndrome coronavirus 2 (SARS-CoV-2) in the country. The number of cases in Italy increased rapidly and the country became the first in Europe to experience a SARS-CoV-2 outbreak.

**Aim:**

Our aim was to describe the epidemiology and transmission dynamics of the first COVID-19 cases in Italy amid ongoing control measures.

**Methods:**

We analysed all RT-PCR-confirmed COVID-19 cases reported to the national integrated surveillance system until 31 March 2020. We provide a descriptive epidemiological summary and estimate the basic and net reproductive numbers by region.

**Results:**

Of the 98,716 cases of COVID-19 analysed, 9,512 were healthcare workers. Of the 10,943 reported COVID-19-associated deaths (crude case fatality ratio: 11.1%) 49.5% occurred in cases older than 80 years. Male sex and age were independent risk factors for COVID-19 death. Estimates of R_0_ varied between 2.50 (95% confidence interval (CI): 2.18–2.83) in Tuscany and 3.00 (95% CI: 2.68–3.33) in Lazio. The net reproduction number R_t_ in northern regions started decreasing immediately after the first detection.

**Conclusion:**

The COVID-19 outbreak in Italy showed a clustering onset similar to the one in Wuhan, China. R_0_ at 2.96 in Lombardy combined with delayed detection explains the high case load and rapid geographical spread. Overall, R_t_ in Italian regions showed early signs of decrease, with large diversity in incidence, supporting the importance of combined non-pharmacological control measures.

## Introduction

Severe acute respiratory syndrome coronavirus 2 (SARS-CoV-2) infection in humans causing clusters of severe pneumonia [1-3] was first detected in the city of Wuhan, China, in December 2019 and designated as SARS-CoV-2 based on phylogeny, taxonomy and established practice [4]. Although of probable zoonotic origin, human-to-human transmission rapidly fuelled the spread of SARS-CoV-2 infections globally; the main known route of transmission is through respiratory droplets, however air-borne transmission has been acknowledged in specific settings (e.g. aerosol-generating medical procedures) [5]. Transmission through contact with contaminated objects/surfaces (fomite transmission) is considered possible. 

Cases in Europe were initially limited to small travel-related clusters in Germany [6], France [6,7] and the United Kingdom [8]. On 20 February 2020, the first case of locally acquired SARS-CoV-2 infection was diagnosed in northern Italy in a critically ill, hospitalised young man with no travel history to known areas of viral circulation or link to a probable or confirmed case of coronavirus disease (COVID-19). Before this date, only three cases of COVID-19 had been reported in central Italy, all with a travel history to Wuhan [9].

Following this unexpected finding, extensive contact tracing and testing of close contacts revealed ongoing transmission in several municipalities of the Lombardy region [10,11]. In subsequent days and weeks, case counts and death tolls increased rapidly, at first in northern Italy and then in the rest of the country. The Italian government imposed increasingly strict physical distancing measures starting with the closure of 10 municipalities in the Lodi province (Lombardy) and one in the Padua province (Veneto) on 23 February 2020. This culminated in a national lockdown on 11 March 2020 [12,13].

The aim of this study was to describe the epidemiology and transmission dynamics of the first COVID-19 cases in Italy in the context of the progressive implementation of control measures culminating in a national lockdown. We summarise the key epidemiological findings from the first 98,716 confirmed COVID-19 cases in Italy, including 10,943 associated deaths, assess risk factors independently associated with death and analyse basic and net reproductive numbers by symptom onset across different regions to explore early SARS-CoV-2 transmission trends.

## Methods

A case-based surveillance system for all laboratory-confirmed human SARS-CoV-2 infections, following the case definition from the European Centre for Disease Prevention and Control (ECDC) [14], was established on 27 February 2020. Before that date, surveillance had focused, also in line with European Union (EU) recommendations, on suspected and confirmed COVID-19 severe respiratory disease [9]. Data were collected daily from the 21 Italian regions and autonomous provinces (AP), using a secure online platform. In the early phases of the outbreak, if uploading to the secure platform was not possible, regions and AP sent datasets daily via email. The following information was collected: demographics, clinical severity, comorbidities, date of symptom onset, date of diagnosis, outcome, region of diagnosis and province of residence. Data on the three imported cases that were notified before 20 February were integrated in the new database.

Laboratory confirmation by RT-PCR was performed on nasopharyngeal swabs as previously described [11,15]. From the beginning of the outbreak until 1 March 2020, all initially confirmed cases were sent to the National Reference Laboratory in the Istituto Superiore di Sanità (ISS) and re-confirmed using RT-PCR protocols that were based on the methods described by Corman et al. [16] and the United States Centers for Disease Control and Prevention (US CDC) [17]. Concordance among confirmation results of 99% demonstrated confirmation capacity of the regional laboratory network. As a consequence, after 1 March, all COVID-19 cases were confirmed directly by regional reference laboratories [18].

Every day, the data were harmonised in a single dataset, cleaned and analysed to produce the main surveillance outputs (infographics and detailed bulletins). These outputs are publicly available on the ISS epidemiology web portal (EpiCentro) [19].

We extracted consolidated data from the integrated surveillance system on 31 March 2020 and integrated imported cases confirmed before 27 February and notified to the previous surveillance system.

We summarised the data by age group and sex and counted cases by date of diagnosis/sample and of symptom onset. We aggregated cases by region/AP of diagnosis and by municipality of residence for cases residing in the same region/AP of diagnosis. Attack rates per 100,000 population by region/AP were calculated using population estimates for 2019, available from the Italian National Institute of Statistics (Istituto Nazionale di Statistica; ISTAT) and adjusted using the age distribution of the Italian population as a reference. We classified the attack rates in each region as high, intermediate and low based on the interquartile range (IQR) of the adjusted attack rates as follows: (i) high: attack rates higher than the upper limit of the IQR, (ii) intermediate: within the IQR, (iii) low: lower than the lower limit of the IQR.

Case fatality ratios (CFR) were calculated by age and sex and smoothed with the locally weighted regression method. Data were analysed as per the latest available data extracted, not adjusting for the reporting delay that is expected to affect more recent data. As the epidemic was still evolving at the time this analysis was performed, we decided to perform minimum group-level adjustments. The crude odds ratios (OR) of death by age were also calculated by calendar period of diagnosis/sample by identifying the following calendar week periods: before 4 March (when data was sparse), 4–10, 11–17, 18–24 and 25–31 March. We also assessed the OR of death among HCW. A multilevel (clustered by region/AP) multivariable logistic model was applied to evaluate characteristics associated with death, including age group (i.e. < 40, 40–49, 50–59, 60–69, 70–79, 80–89, ≥ 90 years), sex, HCW status and week of diagnosis/sample. Crude and adjusted odds ratios were estimated. 

The analyses were performed using STATA (version 16) and R (version 3.6.3). The list of the R packages used for the analysis is available in the Supplement.

### Definitions

Clinical severity for people with laboratory-confirmed SARS-CoV-2 infection was defined as follows: (i) asymptomatic: no apparent signs or symptoms of disease, (ii) paucisymptomatic: general mild symptoms (e.g. general malaise, low grade fever, tiredness) but no clear signs of disease, (iii) mild: clear signs and symptoms of disease (e.g. symptoms of respiratory disease described in the case definition as dry cough and shortness of breath) but not severe enough to require hospitalisation, (iv) severe: clear signs and symptoms of disease (e.g. respiratory disease) and severe enough to require hospitalisation, and (v) critical: clear signs and symptoms of disease (e.g. respiratory disease) and severe enough to require admission to an intensive care unit (ICU).

The surveillance system captures whether the reported subject is a healthcare worker (HCW). We defined a HCW broadly as a person who has ever worked in the healthcare sector regardless of role, profession or current working status. The system also records whether the affected person has one of the following comorbidities: cardio-vascular diseases, respiratory diseases, diabetes, immunodeficiencies, metabolic diseases, oncological diseases, obesity, kidney diseases or other chronic diseases.

We defined a COVID-19-associated death as any person with a laboratory-confirmed SARS-CoV-2 infection who has died, regardless of where the death occurred (hospital, home, other). This definition did not include a temporal or causal component.

### Transmission dynamics

The basic reproduction number R_0_ is defined as the average number of secondary cases generated in a fully susceptible population by a primary infector. This is an expression of the potential for transmission in the absence of any containment measure. However, once interventions are introduced or the susceptibility in the population decreases, the transmission potential at a given time t is measured as the net reproduction number *R_t_*. In this paper, we estimated both R_0_ and R_t_ for Italian regions in different epidemiological situations (high and intermediate age-adjusted attack rates), selected among those with highest data robustness. We used a previously described Bayesian approach [20-22], informed by estimates of the serial interval distribution (average: 6.6 days) from contact tracing data in Lombardy [11]. Details are reported in the Supplement. Modelling was based on data available by 31 March 2020.

### Ethical statement

This study was conducted using data from the Italian national integrated COVID-19 surveillance routinely collected and analysed within the mandate of the Italian National Institute of Health; therefore, no ethical approval was necessary.

## Results

**Figure 1:**
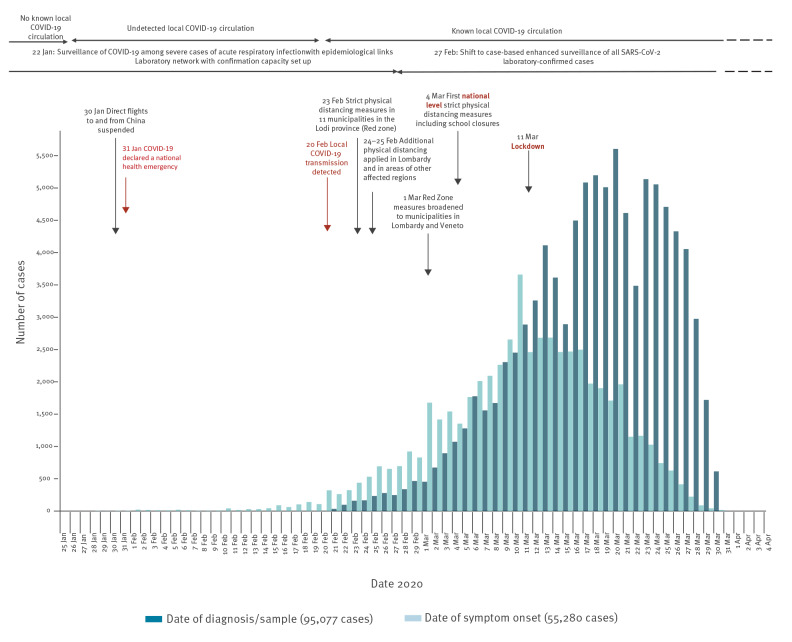
Epidemiological curves of COVID-19 cases by date of onset and date of diagnosis/sample, Italy, 28 January–31 March 2020 (n = 98,716)

COVID-19: coronavirus disease; SARS-CoV-2: severe acute respiratory syndrome coronavirus 2.

From 28 January (when the first imported case was detected) to 31 March 2020 (date of data extraction), 98,716 confirmed cases of COVID-19 were reported including 10,943 related deaths. After a rapid increase, the number of reported cases began to stabilise and decrease (Figure 1). Locally acquired cases diagnosed from the end of February reported onset of symptoms from 28 January onwards (819 cases with symptom onset before 20 February), indicating undetected local transmission for at least 3 weeks before detection. The average delay between symptom onset and diagnosis/sample in the first month of the outbreak was 5.6 days (median: 5 days, IQR: 2–8 days).

In total 819 cases reported onset of symptoms before 20 February.

Completeness of information by date is not 100%. In total, 2,200 cases reported in the study period are not included in the graph as both dates of symptom onset and date of sample/diagnosis were missing.

Source: Italian National Integrated Surveillance for COVID-19 (updated on 31 March 2020).

Specific control measures were applied nationwide from 4 March. Before that date, distancing measures were in place only in areas of Lombardy and Veneto. On 4 March, the first government decree on a national scale was issued. This introduced measures including suspension of congresses and large meetings, shows (live and cinema), sports events/trainings with audience and any other events that could lead to mass gatherings, closure of all schools and suspension of school trips, visiting restrictions in hospitals and long-term care facilities. It promoted information sharing and increased hygiene measures (e.g. on public transports) allowing other activities to continue provided distancing could be guaranteed. It opened to the possibility of applying agile working/home working where possible through a simplified administrative procedure without imposing it and invited more fragile population groups (e.g. elderly and immunocompromised people) to stay at home as much as possible or at least try to avoid gatherings. On 11 March, another government decree was issued that first imposed more strict physical distancing measures (labelled diffusely as ‘lockdown measures’ in the country) including the suspension of all commercial activities except for those selling primary goods including groceries and drugs. It further imposed the closure of restaurants/bars (allowing some exceptions such as take-away and home delivery) and of services such as hairdressers and beauty salons. It also identified core services and activities that should not be suspended but should apply strict security protocols on site and use home working to the maximum extent, also incentivising the use of leaves and furlough schemes. Movement limitation for productive activities was also introduced at this stage, with subsequent decrees expanded to include all movements within the country, ultimately leading to a general stay-at-home recommendation.

**Figure 2:**
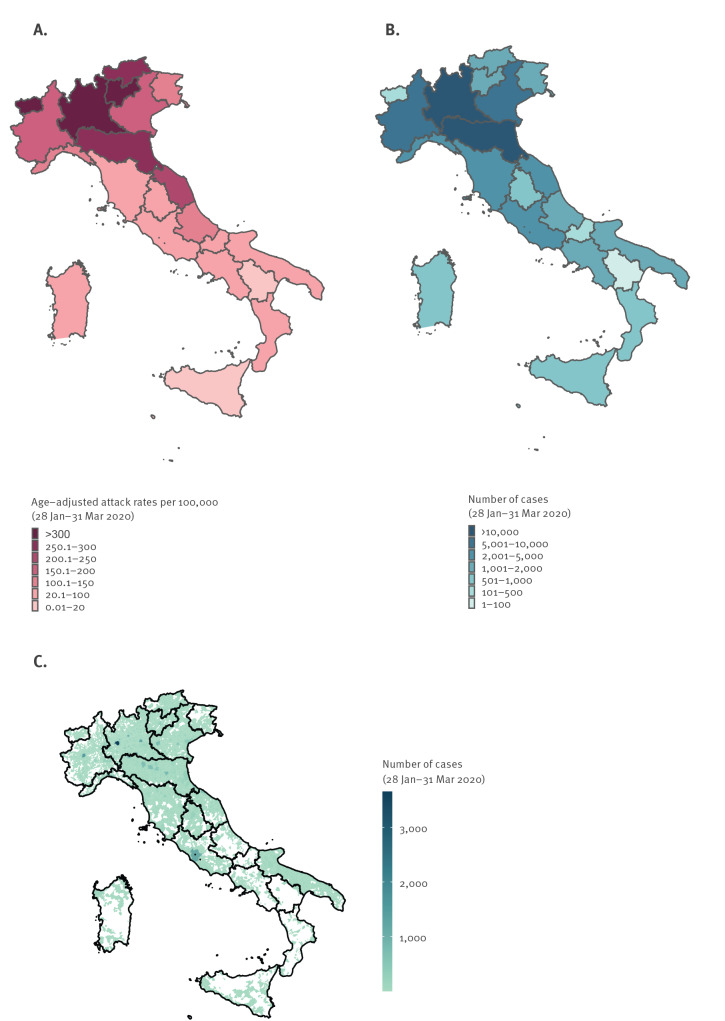
COVID-19 attack rates per 100,000 population (age-adjusted) by region/AP of diagnosis (A) and number of cases by region/AP of diagnosis (n = 98,716) (B) and by municipality of residence when in the region of diagnosis (n = 93,885) (C), Italy, 28 January–31 March 2020

AP: autonomous provinces; COVID-19: coronavirus disease.

Source: Italian National Integrated Surveillance for COVID−19 (updated on 31 March 2020).

By 31 March, 2020, all of Italy’s 21 regions and AP had reported at least one locally acquired case of COVID-19. The country had high incidence areas with sustained local transmission (mainly in the north), low incidence areas with limited but growing numbers of locally acquired cases of infection and regions with intermediate incidence (Figure 2). Overall, 98% (42,279/43,206) of COVID-19 cases diagnosed in Lombardy were among people residing in this region. Among the remaining 2% of cases for whom the place of residence was known, most (123 cases) reported being residents in the neighbouring region of Emilia-Romagna. The index case of the outbreak was not found, and no clear chains of transmission were identified linking initial cases in newly affected regions/AP.

Age-adjusted attack rates per 100,000 were classified as high in Lombardy (crude attack rate: 429.3; age-adjusted attack rate: 431.6/100,000), in the autonomous province of Trento, in Aosta Valley, in the region of Emilia-Romagna and in the autonomous province of Bolzano. Age-adjusted attack rates were classified as intermediate in Marche, Veneto, Piedmont, Liguria, Friuli-Venezia Giulia, Abruzzo, Tuscany, Umbria, Lazio and Apulia. In Molise, Sardinia, Calabria, Campania, Sicily and Basilicata, age-adjusted attack rates were classified as low (Figure 2 and Table 1, Supplementary Table S1).

**Table 1:** Adjusted odds ratios of death in COVID-19 cases reported to national surveillance, Italy, 28 January–31 March 2020 (n = 97,942)

		Crude OR	95% CI	p value	AOR	95% CI	p value
Age (years)	< 40	1.00	Reference		1.00	Reference	
	40–49	3.16	2.05–4.85	< 0.001	3.27	2.12–5.02	< 0.001
	50–59	9.02	6.11–13.33	< 0.001	8.49	5.74–12.55	< 0.001
	60–69	33.16	22.63–48.59	< 0.001	26.29	17.93–38.56	< 0.001
	70–79	103.62	70.89–151.44	< 0.001	80.75	55.20–118.13	< 0.001
	80–89	173.32	118.59–253.30	< 0.001	158.00	107.99–231.17	< 0.001
	≥ 90	174.27	118.61–256.04	< 0.001	202.29	137.42–297.78	< 0.001
Sex	Male vs female	1.85	1.77–1.93	< 0.001	1.85	1.76–1.94	< 0.001
Healthcare worker	Yes vs no/not indicated	0.02	0.01–0.03	< 0.001	0.12	0.08–0.17	< 0.001
Calendar week period of diagnosis (as described in the Methods)	Per 1 week increase	0.64	0.63–0.65	< 0.001	0.59	0.57–0.60	< 0.001

AOR: adjusted odds ratio; CI: confidence interval; OR: odds ratio.

This analysis includes 97,942 of 98,716 (99.2%) cases. The remaining 774 cases were excluded because data on age, sex and/or calendar week were missing. AOR were calculated from a multilevel logistic model clustered on reporting regions/autonomous provinces.

Source: Italian National Integrated Surveillance for COVID−19 (extracted on 31 March 2020).

Most affected cases were male (55.4%) and the median age of cases was 62 years. Notably, 9,512 cases were reported among HCW (median age: 49 years; IQR: 40–59; 33.6% male).

Among 98,503 cases with known age, clinical severity was reported for 35,692 cases. Additional information from sensitivity analyses can be found in the Supplement. In particular, Supplementary Figure 2A shows that the proportion of severe cases younger than 7 years was below 15%. This proportion decreased to 4.9% in the age group 7–19 years. In older age groups, the proportion increased gradually to 33.1% in the age group 80–89 years. Critical severity was reported in cases 20 years and older, reaching 5.5% in the 60–69 year age segment (Supplementary Table S2).

Among all cases, 54,808 (55.5%) were reported to have been managed in their place of residence and 16,625 (16.8%) were hospitalised. For 27,283 (27.6%) cases, this information was not available. As shown in Supplementary Figure 2B, the proportion of hospitalised COVID-19 cases decreased from the 0–1 to the 7–19 year age group and increased progressively from the 20–29 to the 70–79 year age group when it appeared to stabilise. Supplementary Figure 2C shows for all reported hospitalised COVID-19 cases with known unit of admission and age (n = 13,390) the proportion of patients admitted to ICU vs those admitted to any other hospital unit. Overall, the ICU admission rate was 19.2% considering as denominator only cases for whom admission hospital unit was reported. This decreased to 15.5% when all cases reported to have been hospitalised were included in the denominator. There were ICU admissions from the age group 2–6 years onwards, increasing in proportion until age group 60–69 years. The proportion decreased in older age groups.

Of the 10,943 reported COVID-19 associated deaths, 49.5% occurred in cases 80 years or older with an overall crude CFR of 11.1% (the detailed distribution of COVID-19 cases and related deaths reported until 31 March 2020, by age decade and sex are available in Supplementary Table S3). Overall, 21 deaths were reported in HCW (CFR: 0.2%).

**Figure 3:**
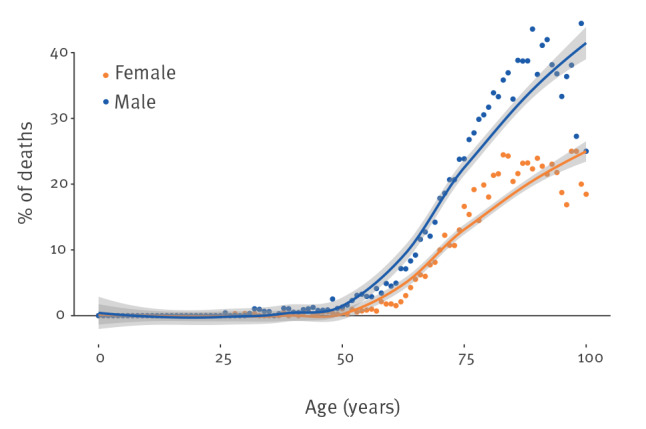
COVID-19 case fatality ratio by age at diagnosis and sex, Italy, 28 January–31 March 2020 (n = 10,940)

Until 31 March 2020, no deaths had been reported among cases younger than 30 years. Overall, 65.6% of the people who died had at least one co-morbidity. Figure 3 shows the CFR reported by 31 March 2020 by single year of age of diagnosed cases stratified by sex smoothed using locally weighted regression curves. There was a sharp increase of the CFR with age in both sexes, however, women had a lower CFR at each age point.

COVID-19: coronavirus disease.

Dots: single years of age; lines: locally weighted regression curves showing the estimated trend; grey shaded area: band width represents the 95% confidence intervals.

Source: Italian National Integrated Surveillance for COVID−19 (updated on 31 March 2020). Three deaths among cases with missing age are not included.

When stratifying by age group and calendar period of diagnosis/sample, we confirmed the age effect on the CFR. Moreover, the CFR calculated for cases diagnosed in the earlier phase of the epidemic and thus with a long follow-up at the time of data extraction was higher, with an overall CFR of 21.9% for people diagnosed with COVID-19 before 4 March (Supplementary Table S4).

After adjusting for age, sex, HCW profession and calendar period of diagnosis/sample, we estimated higher adjusted odds ratios (AOR) of death with increasing age decade and a higher AOR for male compared with female cases. The HCW diagnosed with COVID-19 had lower AOR of death than the non-HCW (Table 1). No evidence of improved fit (p = 0.09, log-likelihood ratio test) was found when testing for possible interaction between sex and age group. We saw similar results when restricting the analysis to 20–70 year-old cases and when excluding from the analysis cases with uncertain HCW status (data not shown). In this further analysis we also evaluated the interaction between age and sex with HCW but again there was not a significant better fit (p = 0.52 for age, p = 0.24 for sex by log-likelihood ratio test).

We estimated the transmission patterns for six Italian regions (Lombardy, Veneto, Emilia-Romagna, Tuscany, Lazio and Apulia) with adjusted AR classification ranging from intermediate to high. These six regions were characterised by different epidemic trajectories. Although with overlapping confidence intervals (CI), variability was also clearly visible in terms of epidemic doubling time, which varied between 2.7 days (95% CI: 2.3–3.3) in Emilia-Romagna and 3.2 days (95% CI: 2.5–4.2) in Veneto (Table 2), and basic reproductive numbers, which were in the range 2.13–3.33.

**Table 2:** Estimated epidemic doubling time and R_0_ in selected regions, Italy, 28 January–31 March 2020 (n = 43,625 cases with a date of symptom onset)

Region	Adjusted AR classification	Doubling time (days)	95% CI	R_0_	95% CI
Lombardy	High	2.7	2.2–3.5	2.96	2.73–3.17
Veneto	Intermediate	3.2	2.5–4.2	2.51	2.18–2.86
Emilia-Romagna	High	2.7	2.3–3.3	2.84	2.57–3.13
Tuscany	Intermediate	3.2	2.3–5.2	2.50	2.18–2.83
Lazio	Intermediate	2.9	2.2–4.3	3.00	2.68–3.33
Apulia	Intermediate	2.9	2.2–4.3	2.61	2.13–3.13

AR: attack rate; CI: confidence interval; R_0_: basic reproduction number.

**Figure 4:**
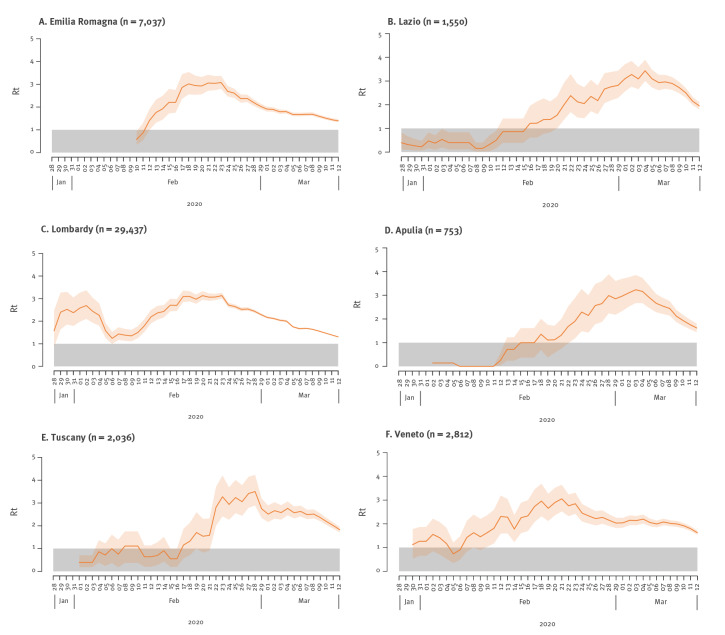
COVID-19 estimated R_t_ in selected Italian regions over a 7-day moving average, Italy, January 28–March 12, 2020

In Lombardy, we estimated that the net reproduction number R_t_ had been above the epidemic threshold since late January 2020 (Figure 4). In February, the R_t_ started to fluctuate, reaching maximum values around 3 in the week from 17 to 23 February. Starting from 24 February, with the enforcement of a quarantined area around the most affected municipalities of the region, R_t_ estimates followed a constantly decreasing trend. The second and third most affected regions in February (Veneto and Emilia-Romagna) showed an increasing trend of R_t_ until about 24 February (Figure 4). At that time, a few tens of cases had been detected in those regions.

COVID-19: coronavirus disease; R_t_: net reproduction number.

Shaded areas: 95% confidence intervals; grey bar: area below the epidemic threshold.

The transmission patterns of Tuscany, Lazio and Apulia were markedly different. In central and southern Italy where these regions are located, the epidemic spread was largely undetected until early March. After an initial increase, R_t_ remained nearly constant at values around 2.5–3 until 4–8 March when physical distancing measures were implemented at national level (Figure 4).

## Discussion

The early epidemiology of COVID-19 in Italy was heterogeneous, with a north-to-south incidence gradient and very different transmission dynamics. However, across all studies regions, transmission consistently decreased following disease detection and implementation of combined non-pharmacological measures. Most of the cases detected in the first month of the outbreak were male with a median age of 62 years. The median age was very high, considering the subsequent evolution of the epidemic [19] in Italy. This influenced the disease outcomes we observed, as the proportion of severe cases increased with age, as did the hospitalisation rate. We also found older age and male sex to be independent risk factors for death associated with COVID-19. 

COVID-19 emerged in Italy with a similar clustering onset as described in Wuhan, China [2], with three major clusters around the cities of Codogno, Bergamo and Cremona in the Lombardy region in northern Italy [11]. Subsequently, cases spread across the country with more sustained transmission in neighbouring regions in the north and in the central region of Marche. Our quantification of the transmission dynamics of the first COVID-19 cases in Italy confirm this observation, with a heterogeneous estimate for the epidemic doubling times and basic reproductive numbers. We also observed varying timing and magnitude of the net reproduction numbers across regions with earlier increases in Lombardy and Veneto and a later onset in central and southern Italy.

The epidemic curve suggests that the earliest cases were detected with a considerable delay (up to 3 weeks), suggesting that the detection of local transmission of SARS-CoV-2 occurred in a context of ongoing transmission. This was later corroborated by environmental studies that highlighted viral circulation in northern Italy at the end of 2019 [23]. The high R_0_, the short serial interval and delayed detection amid ongoing transmission, explain the rapid case increase and geographical spread of the disease in Italy. They also explain the inability to identify the index case and clearly trace the initial spread of infection across the country. 

In all regions, we consistently observed a rapid reduction of the R_t_ after the implementation of control measures. Notably, in northern regions (Lombardy, Veneto and Emilia-Romagna), this marked decreasing trend started immediately after detection of the first case in late February and was possibly due to increased awareness in the population and to the effect of early interventions. After 11 March, lockdown allowed the reproduction number to fall below the epidemic threshold and control the first wave of SARS-CoV-2 in Italy [24].

HCW were disproportionally affected during the first acute phase of the SARS-CoV-2 epidemic in Italy. This underlines the fact that SARS-CoV-2 can easily spread in healthcare contexts and emphasises the importance of strong infection prevention and control practices. In Italy, as described in other countries [25], nursing homes and long-term care facilities have emerged as particularly fragile environments in which infection can spread very rapidly with potentially disastrous outcomes owing to the vulnerability of the hosted populations [26].

As demonstrated previously in China [27], we showed in this study worse outcomes in older male patients with comorbidities. Male sex and older age were independent risk factors for COVID-19-related death, also after adjusting for age, sex, HCW profession and calendar period of diagnosis/sample. HCW diagnosed with COVID-19 had lower AOR of death than non-HCW. Affected HCW, compared with the affected general population, were on average younger and more frequently female. Considering the predominance of female professionals of working age in the Italian health sector, this is the distribution that would be expected in professionally exposed groups. Age and sex only explain in part the lower CFR, as the AOR of death was lower among HCW also after adjusting for those variables and might be related to earlier detection and management as well as to a ‘healthy worker effect’ [28].

Data on clinical severity and ICU admission of COVID-19 cases were consistent with observations on the risk of COVID-19-related deaths. The proportion of severe and critical cases increased with age until age 80 years. The slight decrease in the proportion of critical and severe cases, and the number of deaths, in the higher age groups could be due to the demographic structure of the population with a higher female-to-male ratio among older people [29]. Consistently with reported disease severity, the proportion of COVID-19 related ICU admissions increased until the age group 60–69 years and decreased in older ages. Although rare, some ICU admissions were notified in younger age groups, confirming the potential for critical disease also among children and young adults.

In absolute terms, a COVID-19 CFR of 11.1% was documented in the early phase of the outbreak in Italy. This is higher than that what was observed in other countries at the same time. As recently described, this could in part be explained by the demographic structure of the Italian population [30]. However, other aspects such as Italian testing policies at the time that focussed on symptomatic cases, the choice to include only laboratory-confirmed cases and to define associated deaths in a very inclusive manner, may also have played a role in making initial case fatality data poorly comparable across countries. Also, the overall ICU admission rate was much higher than the reported 4% in 16 EU countries as published at the time by the ECDC [31]. We are unable to speculate whether the reasons behind this difference are related to a possible detection bias towards more severe cases, hospitalisation policies and practices, or whether there were other factors at play. 

In particular, higher CFR could also be an expression of the impact that an uncontrolled SARS-CoV-2 transmission has on healthcare and public health services, causing shortage of hospital beds and delays in the diagnosis of new cases. Studies from Lombardy have highlighted the potentially catastrophic effects of COVID-19 on the healthcare system [10,11] and the data presented in this paper show the increasing median time between time of symptom onset and time of diagnosis during the first month of the pandemic. As capacity to track cases and test increased, the time between onset and diagnosis decreased again, however this was not evident in this first month. 

Our study has limitations: Most of the cases detected in the first month of the outbreak were male with a median age of 62 years. The sex ratio balanced out and median age became lower in subsequent phases of the outbreak, with an increased proportion of asymptomatic and mild cases and lower CFR [19]. Considering how older age and male sex were found to be risk factors for disease severity and death, this suggests that in the early phase of the outbreak, more severe cases were more likely to be detected. This could be due to a change in the detection policies that led to the restriction of testing to only symptomatic cases on 27 February [32] as well as to the stress on the healthcare system that needed to prioritise the diagnosis and management of more severe cases. The testing policy was broadened to asymptomatic close contacts and to various screening programmes (e.g. ahead of hospital admission for other causes) following the period examined in this study when Italy entered the transition phase and a test–track–trace strategy was adopted.

Further, the data collected from the Italian integrated COVID-19 surveillance system during the initial phase of the emergency presented some shortcomings, mainly related to lack of completeness. For this reason, some stratifications and analyses are not shown. Specifically, the lack of completeness regarding the presence and type of comorbidities did not allow us to include this in the multivariable analysis of deaths in order to assess, and/or adjust for, this factor. In addition, not all regions reported the date of sampling at the beginning of the outbreak and when missing, we used date of diagnosis to construct epidemic curves. This has limitations because there is a lag between diagnostic sampling and confirmation of laboratory results. However, this interval is expected to be limited to 2–3 days and not to bias excessively the presentation of the time distribution of cases, especially when combined with the epidemic curve by date of symptom onset. We did not adjust data on hospitalisation and ICU admissions as well as CFR for the expected time for disease evolution and may therefore have underestimated it in the more recent period. Finally, the estimation of R_0_, R_t_ and the doubling time were made in regions selected on the basis of the robustness of data considering epidemiologically diverse settings.

Another relevant consideration is that the early phase of the COVID-19 epidemic in Italy was characterised by a large number of cases with a short follow-up time since diagnosis. This implies that some of the notified cases that would die at a later date would still be alive at the time of observation and would therefore not be counted in the CFR. As a consequence, overall CFR collected in the acute epidemic phase of potentially lethal infectious diseases with relatively long time from onset to death might be underestimated. We confirmed that this is also the case for COVID-19 by performing an analysis by period of diagnosis/sample. We found that those diagnosed before 4 March 2020 had an overall CFR of around 20%. Subsequent studies with longer follow-up clarified this aspect better, including studies evaluating overall population excess mortality, which are also more comparable across countries [33-35].

## Conclusion

Even in the presence of the mentioned limitations, our analysis provides an overview of the early epidemiology of the COVID-19 outbreak in Italy, showing how in the context of evident sub-national differences in transmission dynamics, transmission consistently decreased following disease detection and implementation of combined non-pharmacological measures.

## Supplementary Data

1537000 Supplement
