# Supplementary material for: Epidemiological characteristics of COVID-19 cases and estimates of the reproductive numbers 1 month into the epidemic, Italy, 28 January to 31 March 2020
Source: Euro Surveill. 2020 Dec 10;25(49):2000790. doi: 10.2807/1560-7917.ES.2020.25.49.2000790 (PMC7730489; doi:10.2807/1560-7917.ES.2020.25.49.2000790)
Supplement: Supplement [file 20-00790_RICCARDO_supplement.pdf]

Supplementary materials to the article:

Epidemiological characteristics of COVID-19 cases and estimates of the reproductive numbers 1 month into the epidemic, Italy, 28 January to 31 March 2020

This supplementary material is hosted by *Eurosurveillance* as supporting information alongside the article '*Epidemiological characteristics of COVID-19 cases and estimates of the reproductive numbers 1 month into the epidemic, Italy, 28 January to 31 March 2020*', on behalf of the authors, who remain responsible for the accuracy and appropriateness of the content. The same standards for ethics, copyright, attributions and permissions as for the article apply. Supplements are not edited by *Eurosurveillance* and the journal is not responsible for the maintenance of any links or email addresses provided therein.

FIGURE 1 – MAP OF THE 21 ITALIAN REGIONS AND AUTONOMOUS PROVINCES

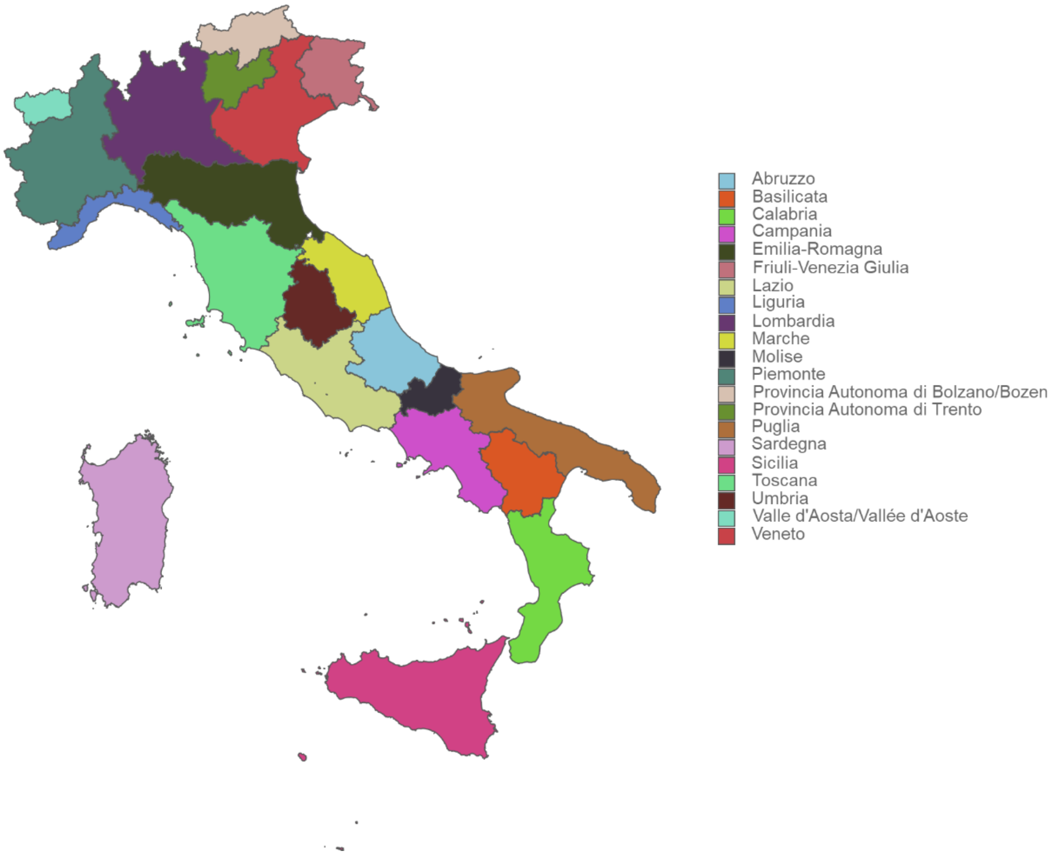

FIGURE 2 – A. REPORTED CLINICAL SEVERITY OF CONFIRMED COVID-19 CASES IN MARCH 31, 2020 IN ITALY (N=35,692). B. REPORTED PROPORTION OF COVID-19 CASES BY PLACE OF RESIDENCE VS HOSPITAL MANAGEMENT (N= 71,365) AND C. PROPORTION OF HOSPITALIZED COVID-19 CASES (N= 13,390) BY ICU VS OTHER UNIT OF HOSPITALISATION, BY AGE GROUP (B), ITALY, 31 MARCH 2020.

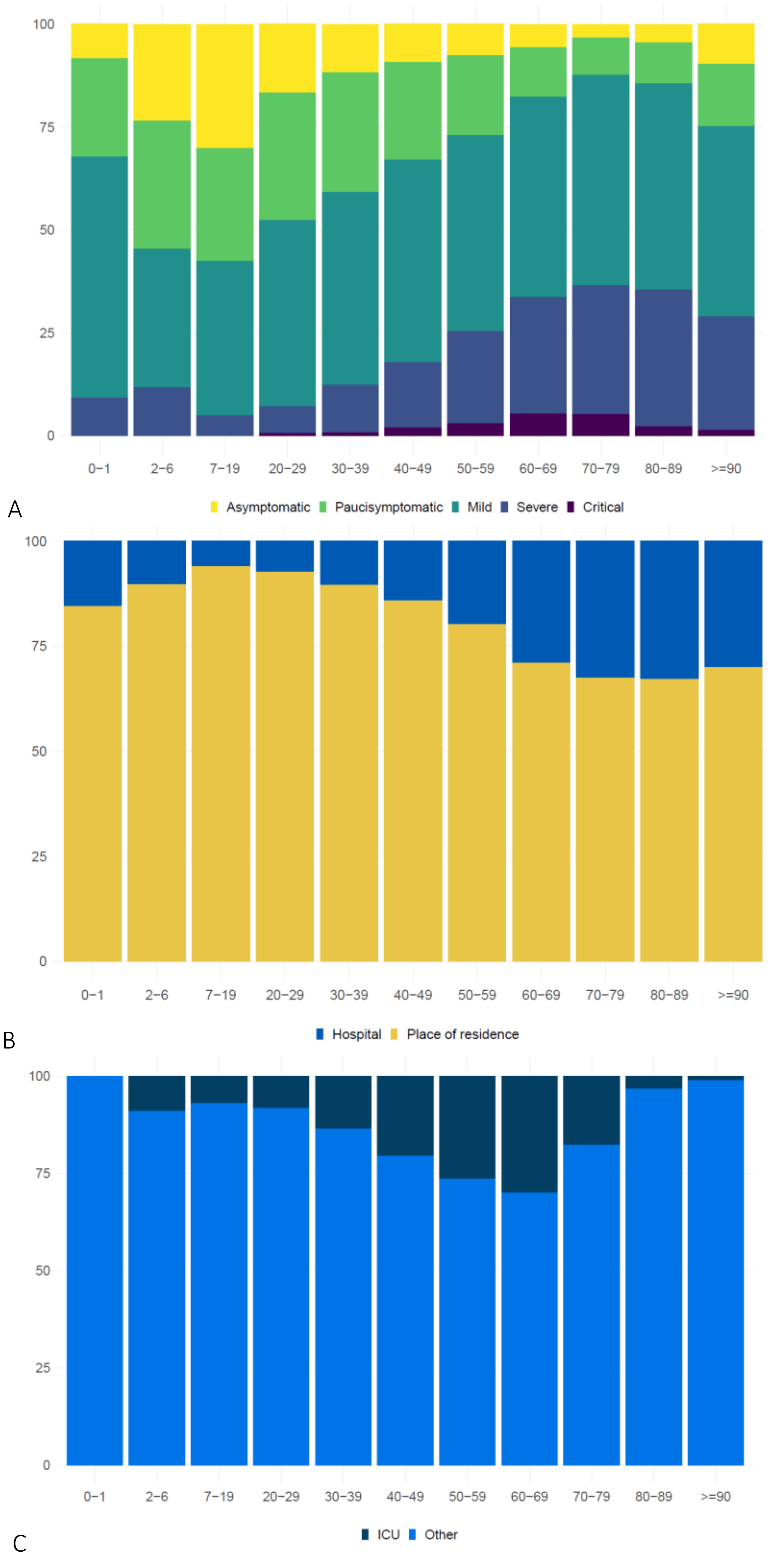

Table 1 - Distribution of diagnosed COVID-19 cases (n=62,843), crude and adjusted Attack Rates, by Region/AP, Italy, 31 March 2020

Region/AP	Total number of cases	Crude attack rates (per 100,000)	Adjusted attack rates (per 100,000)	Adjusted attack rate classification
Lombardia	43206	429.3	431.6	High
Provincia Autonoma di Trento	1853	342.5	349.6	High
Valle d'Aosta/Vallée d'Aoste	447	351.7	346.7	High
Emilia-Romagna	13486	302.4	294.9	High
Provincia Autonoma di Bolzano/Bozen	1371	257.4	274.4	High
Marche	3734	244	233.9	Intermediate
Veneto	9158	186.6	185.9	Intermediate
Piemonte	8104	185.9	177.1	Intermediate
Liguria	2135	136.6	122.5	Intermediate
Friuli-Venezia Giulia	1441	118.3	113.3	Intermediate
Abruzzo	1351	102.6	101	Intermediate
Toscana	3552	95	91.4	Intermediate
Umbria	703	79.5	78.7	Intermediate
Lazio	2910	49.3	49.8	Intermediate
Puglia	1803	44.6	45.6	Intermediate
Molise	143	46.5	45.2	Low
Sardegna	546	33.1	32.1	Low
Calabria	507	25.8	26.4	Low
Campania	1392	23.5	25	Low
Sicilia	828	15.3	15.9	Low
Basilicata	46	8.3	8.2	Low
Total	**98.716**			
Note: cases with missing age (n-203) have been excluded from the estimation of the attack rates				

Table 2 – Reported clinical severity of COVID-19 cases (n=35,692) by age group, Italy, 31 March 2020

Age group	***Asymptomatic***		***Paucisymptomatic***		***Mild***		***Severe***		***Critical***		***Total***
	N	%	N	%	N	%	N	%	N	%	
0-1	11	8.4	31	23.7	77	58.8	12	9.2	0	0	*131*
2-6	16	23.5	21	30.9	23	33.8	8	11.8	0	0	*68*
7-19	104	30.1	94	27.2	130	37.7	17	4.9	0	0	*345*
20-29	222	16.7	411	30.9	603	45.3	85	6.4	10	0.8	*1,331*
30-39	288	11.7	717	29	1,163	47	282	11.4	22	0.9	*2,472*
40-49	433	9.3	1,097	23.6	2,302	49.4	729	15.7	96	2.1	*4,657*
50-59	607	7.7	1,506	19.2	3,760	47.9	1,741	22.2	242	3.1	*7,856*
60-69	391	5.5	848	12	3,438	48.7	1,987	28.2	391	5.5	*7,055*
70-79	216	3.2	598	8.9	3,430	51.3	2,086	31.2	356	5.3	*6,686*
80-89	192	4.5	416	9.7	2,147	50.3	1,413	33.1	101	2.4	*4,269*
>=90	80	9.7	123	15	382	46.5	225	27.4	12	1.5	*822*
Total	*2,560*		*5,862*		*17,455*		*8,585*		*1,230*		*35,692*

Table 3 - Distribution of COVID-19 cases (n=98,716) and related deaths (n=10,943) reported until March 31, 2020, by age and sex

Age groups	Males					Females					Total cases				
	N. Cases	% Cases by sex	N. Deaths	% Deaths by sex	% CFR	N. Cases	% Cases by sex	N. Deaths	% Deaths by sex	% CFR	N. Cases	% Cases by age group	N. Deaths	% Deaths by age group	% CFR
0-9	335	55.6	0	0.0	0.0	267	44.4	0	0.0	0.0	607	0.6	0	0.0	0.0
10-19	409	50.9	0	0.0	0.0	395	49.1	0	0.0	0.0	807	0.8	0	0.0	0.0
20-29	1,724	43.3	1	50.0	0.1	2,258	56.7	1	50.0	0.0	4,049	4.1	2	0.0	0.0
30-39	3,223	47.7	20	80.0	0.6	3,536	52.3	5	20.0	0.1	6,827	6.9	25	0.2	0.4
40-49	6,063	48.4	67	72.8	1.1	6,475	51.6	25	27.2	0.4	12,625	12.8	92	0.8	0.7
50-59	10,478	54.0	321	78.7	3.1	8,912	46.0	87	21.3	1.0	19,505	19.8	409	3.7	2.1
60-69	11,181	65.8	1,013	79.9	9.1	5,823	34.2	255	20.1	4.4	17,074	17.3	1,271	11.6	7.4
70-79	11,662	64.5	2,791	75.1	23.9	6,426	35.5	927	24.9	14.4	18,174	18.4	3,724	34.0	20.5
80-89	7,986	53.5	2,840	65.1	35.6	6,936	46.5	1,523	34.9	22.0	14,980	15.2	4,366	39.9	29.1
≥90	1,242	32.2	475	45.2	38.2	2,611	67.8	575	54.8	22.0	3,865	3.9	1,051	9.6	27.2
Not reported	105	52.5	3	100.0	2.9	95	47.5	0	0.0	0.0	203	0.2	3	0.0	1.5
Total	54,408		7531		13.8	43,734		3398		7.8	98,716		10943		11.1

Table 4. Case fatality rate by age group and calendar period of COVID-19 diagnosis

	Calendar period of diagnosis													
	≤March 3			March 4-10			March 11-17			March 18-24			March 25-31	
**Age class**	**Cases**	**Deaths**	**CFR**	**Cases**	**Deaths**	**CFR**	**Cases**	**Deaths**	**CFR**	**Cases**	**Deaths**	**CFR**	**Cases**	**Deaths**
**<40**	507	0	0.0	1,338	10	0.7	3,318	11	0.3	4,137	6	0.1	2,990	0
**40-49**	460	1	0.2	1,408	26	1.8	3,440	36	1.0	4,392	23	0.5	2,925	6
**50-59**	746	30	4.0	2,301	94	4.1	5,098	162	3.2	7,025	96	1.4	4,335	27
**60-69**	712	115	16.2	2,220	268	12.1	4,692	494	10.5	6,102	321	5.3	3,348	73
**70-79**	863	341	39.5	2,578	858	33.3	5,083	1359	26.7	6,096	984	16.1	3,554	182
**80-89**	649	327	50.4	1,897	940	49.6	3,831	1544	40.3	5,005	1197	23.9	3,598	358
**90+**	113	71	62.8	360	205	56.9	838	344	41.1	1329	315	23.7	1225	116
**Total**	4,050	885	21.9	12,102	2,401	19.8	26,300	3,950	15.0	34,086	2,942	8.6	21,975	762

98,513/98,716 cases included in this table

ESTIMATION OF THE REPRODUCTION NUMBER

The basic reproduction number R_0 represents the average number of secondary cases generated by a primary infector in a fully susceptible population. In general terms, when R_0 is larger than 1 the infection may spread in the population and the larger R_0 the larger effort required to control the epidemic. Once the number of susceptible individuals declines, the transmission potential of the disease at a given time t is measured in terms of the net reproduction number R(t). The net reproduction number is useful to track the effectiveness of performed control measures and other factors affecting the spread of the epidemic (e.g., the behavioral response of the population) over time. As soon as R(t) falls below 1, the epidemic starts to decline.

To estimate R(t), we use the same methodology presented in reference [1-3]. We assumed that the daily number of new cases (date of symptom onset) with locally acquired infection L(t) can be approximated by a Poisson distribution according to the equation

$$L(t) \sim \mathrm{Pois}\left(R(t)\sum_{s=0}^{t}\varphi(s)C(t-s)\right)$$

where

- C(t), with t from 1 to T, is daily number of new cases (date of symptom onset);
- R(t) is the net reproduction number at time t;
- $\varphi(s)$ is the distribution of the generation time (corresponding to the distribution of the serial interval) calculated at time s. From the analysis of 90 observations of individual serial intervals in 55 clusters, the distribution of the serial interval was estimated to follow a gamma distribution with mean 6.6 days (percentiles 2.5th and 97.5th of the distribution: 0.7-19.0) [4].

The likelihood $\mathcal{L}$ of the observed time series of cases from day 1 to day T conditional on C(0) is thus given by

$$\mathcal{L} = \prod_{t=1}^{T} P\left(L(t);\ R(t)\sum_{s=1}^{t}\varphi(s)C(t-s))\right)$$

where P(k; λ) is the probability mass function of a Poisson distribution (i.e., the probability of observing k events if these events occur with rate λ).

We then used MCMC Metropolis-Hastings sampling to estimate the posterior distribution of R(t). To estimate R_0, we assumed that during the period where the epidemic showed exponential growth R(t)=R_0 and used the above described procedure.

We assessed robustness of data on the basis of both data completeness and number of cases for statistical power. In particular, for the study we selected those regions that had that confirmed at least 1,000 cases by March 24 and reported a date of symptom onset for at least 40% of the cumulative confirmed cases. Because Veneto, unlike all other selected regions, included asymptomatic individuals in the testing criteria, we included this region despite the fact that the percentage of confirmed cases with a date of symptom onset was lower (33%) than the considered threshold.

REFERENCES

1. World Health Organization Ebola Response Team, Ebola virus disease in West Africa--the first 9 months of the epidemic and forward projections. N Engl J Med 2014; 371(16): 1481-95.

2. Liu Q-H, et al. Measurability of the epidemic reproduction number in data-driven contact networks. Proc Natl Acad Sci USA 2018; 115(50): 12680.

3. Zhang J, et al. Evolving epidemiology of novel coronavirus diseases 2019 and possible interruption of local transmission outside Hubei Province in China: a descriptive and modeling study. medRxiv 2020; doi: https://doi.org/10.1101/2020.02.21.20026328

4. Cereda D, et al. The early phase of the COVID-19 outbreak in Lombardy, Italy. 23 Mar 2020. Available from: https://arxiv.org/abs/2003.09320

REFERENCES OF THE R PACKAGES USED

Achim Zeileis, Yves Croissant (2010). Extended Model Formulas in R: Multiple Parts and Multiple Responses. Journal of Statistical Software 34(1), 1-13. doi:10.18637/jss.v034.i01

Alastair Rushworth (2019). inspectdf: Inspection, Comparison and Visualisation of Data Frames. R package version 0.0.7. https://CRAN.R-project.org/package=inspectdf

David Gohel (2020). officer: Manipulation of Microsoft Word and PowerPoint Documents. R package version 0.3.8. https://CRAN.R-project.org/package=officer

David Gohel (2020). rvg: R Graphics Devices for Vector Graphics Output. R package version 0.2.4. https://CRAN.R-project.org/package=rvg

David Robinson and Alex Hayes (2020). broom: Convert Statistical Analysis Objects into Tidy Tibbles. R package version 0.5.5. https://CRAN.R-project.org/package=broom

Frank E Harrell Jr, with contributions from Charles Dupont and many others. (2020). Hmisc: Harrell Miscellaneous. R package version 4.3-1. https://CRAN.R-project.org/package=Hmisc

Garrett Grolemund, Hadley Wickham (2011). Dates and Times Made Easy with lubridate. Journal of Statistical Software, 40(3), 1-25. URL http://www.jstatsoft.org/v40/i03/.

Haddley Wickham. ggplot2: Elegant Graphics for Data Analysis. Springer-Verlag New York, 2016.

Hadley Wickham (2011). The Split-Apply-Combine Strategy for Data Analysis. Journal of Statistical Software, 40(1), 1-29. URL http://www.jstatsoft.org/v40/i01/.

Hadley Wickham (2019). stringr: Simple, Consistent Wrappers for Common String Operations. R package version 1.4.0. https://CRAN.R-project.org/package=stringr

Hadley Wickham (2020). forcats: Tools for Working with Categorical Variables (Factors). R package version 0.5.0. https://CRAN.R-project.org/package=forcats

Hadley Wickham and Dana Seidel (2019). scales: Scale Functions for Visualization. R package version 1.1.0. https://CRAN.R-project.org/package=scales

Hadley Wickham and Evan Miller (2019). haven: Import and Export 'SPSS', 'Stata' and 'SAS' Files. R package version 2.2.0. https://CRAN.R-project.org/package=haven

Hadley Wickham and Jennifer Bryan (2019). readxl: Read Excel Files. R package version 1.3.1. https://CRAN.R-project.org/package=readxl

Hadley Wickham and Lionel Henry (2020). tidyr: Tidy Messy Data. R package version 1.0.2. https://CRAN.R-project.org/package=tidyr

Hadley Wickham, Jim Hester and Romain Francois (2018). readr: Read Rectangular Text Data. R package version 1.3.1. https://CRAN.R-project.org/package=readr

Hadley Wickham, Romain François, Lionel Henry and Kirill Müller (2020). dplyr: A Grammar of Data Manipulation. R package version 0.8.5. https://CRAN.R-project.org/package=dplyr

Jared E. Knowles (2019). eeptools: Convenience Functions for Education Data. R package version 1.2.2. https://CRAN.R-project.org/package=eeptools

Jim Hester (2020). glue: Interpreted String Literals. R package version 1.3.2. https://CRAN.R-project.org/package=glue

Kamvar ZN, Cai J, Pulliam JRC, Schumacher J, Jombart T. Epidemic curves made easy using the R package incidence [version 1; referees: awaiting peer review]. F1000Research 2019, 8:139. URL https://doi.org/10.12688/f1000research.18002.1.

Kirill Müller and Hadley Wickham (2019). tibble: Simple Data Frames. R package version 2.1.3. https://CRAN.R-project.org/package=tibble

Lionel Henry and Hadley Wickham (2019). purrr: Functional Programming Tools. R package version 0.3.3. https://CRAN.R-project.org/package=purrr

Makowski, D. & Lüdecke, D. (2019). The report package for R: Ensuring the use of best practices for results reporting. CRAN. Available from https://github.com/easystats/report. doi: .

Nowosad, J. (2018). 'CARTOColors' Palettes. R package version 1.0.0. https://nowosad.github.io/rcartocolor

Pebesma, E., 2018. Simple Features for R: Standardized Support for Spatial Vector Data. The R Journal 10 (1), 439-446, https://doi.org/10.32614/RJ-2018-009

Sam Firke (2020). janitor: Simple Tools for Examining and Cleaning Dirty Data. R package version 1.2.1. https://CRAN.R-project.org/package=janitor

Sarkar, Deepayan (2008) Lattice: Multivariate Data Visualization with R. Springer, New York. ISBN 978-0-387-75968-5

Simon Garnier (2018). viridisLite: Default Color Maps from 'matplotlib' (Lite Version). R package version 0.3.0. https://CRAN.R-project.org/package=viridisLite

Therneau T (2015). _A Package for Survival Analysis in S_. version 2.38, https://CRAN.R-project.org/package=survival.

Thomas Lin Pedersen (2019). patchwork: The Composer of Plots. R package version 1.0.0. https://CRAN.R-project.org/package=patchwork

Tomas J. Aragon (2020). epitools: Epidemiology Tools. R package version 0.5-10.1. https://CRAN.R-project.org/package=epitools

Wickham et al., (2019). Welcome to the tidyverse. Journal of Open Source Software, 4(43), 1686, https://doi.org/10.21105/joss.01686

Yihui Xie (2020). knitr: A General-Purpose Package for Dynamic Report Generation in R. R package version 1.28.
